# Variation in One‐Year Mortality Following Severe Weather Exposure Among Older Americans by Chronic Health Condition and Sociodemographic Status

**DOI:** 10.1111/jgs.70237

**Published:** 2025-12-16

**Authors:** Sue Anne Bell, Melissa Fiffer, Jonathan Martindale, Julie P. W. Bynum, Joshua Tootoo, Ryan Zomorrodi, Aaron Lilienfeld, Marie Lynn Miranda, Matthew A. Davis

**Affiliations:** ^1^ School of Nursing, Department of Systems, Populations, and Leadership University of Michigan Ann Arbor Michigan USA; ^2^ Institute for Healthcare Policy and Innovation University of Michigan Ann Arbor Michigan USA; ^3^ Children's Environmental Health Initiative University of Illinois Chicago Chicago Illinois USA; ^4^ School of Medicine University of Michigan Ann Arbor Michigan USA; ^5^ Department of Pediatrics University of Illinois Chicago Chicago Illinois USA; ^6^ Department of Mathematics, Statistics, and Computer Science University of Illinois Chicago Chicago Illinois USA

**Keywords:** chronic disease, dementia, disaster planning, older adults

## Abstract

**Background:**

While the immediate effect of exposure to severe weather from hurricanes on mortality is well documented, it is unknown whether mortality in the year following exposure to severe weather differs across older Americans with specific vulnerable characteristics. This paper sought to determine whether the association between exposure to high rain and one‐year mortality differs across vulnerable subgroups of older adults.

**Methods:**

This retrospective cohort study used Medicare claims data from fee‐for‐service beneficiaries aged ≥ 65 in Texas and Louisiana in the year before and after Hurricane Harvey. Historical weather data was used to construct a 4‐day measure of cumulative rainfall, the primary severe weather caused by Hurricane Harvey. We identified vulnerable subgroups based on five chronic health conditions requiring regular healthcare access, and sociodemographic factors (e.g., ≥ 85 years, dual eligibility). Cox proportional hazards regression was used to adjust for covariates when estimating the association between high rain exposure and mortality up to 1 year after exposure.

**Results:**

In adjusted models, high rain exposure was significantly associated with greater mortality risk (HR 1.03, 95% CI 1.01–1.05). Among those with chronic health conditions including Alzheimer's disease and related dementias (ADRD) (HR 1.05 [95% CI 1.03, 1.08]), diabetes (HR 1.04 [1.02, 1.07]), and chronic kidney disease (HR 1.04 [1.01, 1.06]) exposed to high rain versus those unexposed to high rain, associations with high rain were found. Higher mortality was also observed among Non‐Hispanic Black (HR 1.06 [95% CI 1.01, 1.11]) and Hispanic and Latino populations (HR 1.13 [95% CI 1.08, 1.19]).

**Conclusion:**

Exposure to high rain from Hurricane Harvey was associated with higher one‐year mortality that varied across vulnerable groups. The largest associations were observed among older adults with health conditions that require regular healthcare (e.g., CKD, ADRD) and minoritized racial and ethnic groups.

## Summary

- Key points
  ○ Exposure to high rainfall during Hurricane Harvey was associated with increased one‐year mortality among older adults.
  ○ Mortality risk was highest among those with chronic health conditions requiring regular care, including chronic kidney disease and Alzheimer's disease and related dementias.
  ○ Non‐Hispanic Black and Hispanic/Latino populations experienced higher mortality risks, highlighting disparities in disaster vulnerability.
- Why does this paper matter?
  ○ This study provides new evidence that severe weather events have long‐term health consequences for vulnerable older adults. Identifying populations at heightened risk for disasters can inform targeted preparedness and healthcare interventions to reduce mortality disparities.

## 1 Introduction

In 2023, the United States experienced 28 separate weather and climate disasters with total damages nearing $93 billion and 492 reported deaths [1]. There is an urgent need to understand disaster‐associated mortality from these events, particularly related to hurricane exposure among older adults given their high population growth and concentration in the 18 hurricane‐prone Atlantic and Gulf Coast states [2, 3, 4].

Previous studies have examined mortality due to the direct effects of major storms [5, 6], but considerably less is known about mortality beyond the immediate aftermath of severe weather and the degree to which mortality may differ among older adults with vulnerable characteristics. A better understanding of those groups at highest risk can inform emergency planning that considers the unique needs of specific populations of older adults. In particular, understanding long‐term mortality among older adults with chronic health conditions who have complex healthcare needs or who require caregiving, as well as those facing social and economic vulnerabilities, would allow for improved long‐term recovery planning, including building more resilient and responsive systems of healthcare [7].

Therefore, the objective of this study was to determine whether the association between severe weather—defined as high rainfall, the primary form of severe weather caused by Hurricane Harvey—and mortality varies across older Americans by sociodemographic status and with certain health conditions, including Alzheimer's disease and/or other related dementias (ADRD), congestive heart failure (CHF), diabetes (DM), chronic obstructive pulmonary disease (COPD), and chronic kidney disease (CKD).

## 2 Methods

Using a combination of Medicare administrative claims and historic weather data, we conducted a retrospective cohort study to determine whether the association between high rainfall from Hurricane Harvey and mortality differed across specific characteristics. Vulnerable populations were identified based on five common chronic health conditions reliant on regular access to healthcare as well as conventional sociodemographic characteristics: the oldest old, minoritized racial and ethnic groups, and those dually eligible (i.e., beneficiaries enrolled in both Medicare and Medicaid). This study received an expedited review by the University of Michigan Institutional Review Board and follows STROBE reporting guidelines.

### 2.1 Study Population and Data Source

We used 100% Medicare administrative claims data to identify Fee‐For‐Service (FFS) beneficiaries who resided in either Texas or Louisiana during the year before and after Hurricane Harvey. Texas and Louisiana were selected because they had one or more Federal Emergency Management Administration (FEMA) disaster‐declared counties during Hurricane Harvey—inclusion of the entire states as the study sample helped ensure many unexposed older adults but with shared regional factors that may contribute to mortality risk. Beneficiaries were required to be aged 65 or older and have FFS coverage starting on January 1, 2016, and maintain coverage through the end of August 2017, the month of Harvey's landfall, to ensure proper coverage for identifying those with health conditions of interest using the Master Beneficiary Summary File (MBSF) Chronic Conditions Segment (Figure S1). FFS coverage post‐landfall was not required since our main outcome, date of death, was available for all beneficiaries regardless of Medicare coverage type. Those who relocated during the follow‐up period (*N* = 49,936) were excluded. This resulted in a final sample of 1,781,217 beneficiaries, with 1,468,658 (82.5%) residing in Texas and 312,559 (17.5%) in Louisiana. Beneficiary Zone Improvement Plan (ZIP) codes were converted to ZIP code tabulation areas (ZCTAs) using the 2017 UDS Mapper ZIP Code to ZCTA Crosswalk [8].

#### 2.1.1 Vulnerable Older Adult Populations by Health Condition

The MBSF chronic condition segment was used to identify populations of older adults diagnosed with ADRD, CHF, CKD, COPD, and DM [9, 10]. These health conditions were selected based on literature review and clinical relevance due to their reliance on regular healthcare access or caregiving. Older adults living with these health conditions need access to electrical power in the home, healthful nutrition, transportation to healthcare appointments, and in most cases, a regular and trusted caregiver, all of which may be disrupted during a disaster [11]. For example, breakdowns in kidney disease care in past disasters including Hurricanes Katrina, Sandy and Maria, have resulted in adverse outcomes, including mortality [12, 13, 14, 15, 16, 17, 18].

#### 2.1.2 Vulnerable Older Adult Populations by Sociodemographic Status

Sub‐populations were selected to identify potentially vulnerable groups who may be more significantly impacted by severe weather exposure due to age or racial and ethnic minority status. Enrollment data from the MBSF was used to identify individuals who had any evidence of dual enrollment (in Medicare and Medicaid) in the year prior to landfall, which serves as a surrogate measure of low income. The Research Triangle Institute race measure reported in the MBSF was used to identify individuals from historically marginalized racial and ethnic groups (i.e., Non‐Hispanic Black and Hispanic/Latino populations) [19]. We also identified individuals who were 85 or older (i.e., the “oldest old”).

### 2.2 Exposure to High Rain (Independent Variable)

Past studies of disaster‐related mortality have estimated exposure at the county‐level [7], and relied primarily on either FEMA‐disaster declared county designation, or distance to the central track of a storm [8]. However, examining storm exposures at a more resolved geographic scale (e.g., ZCTA) can reveal variation obscured at the county level. Severe weather can be measured in multiple ways (e.g., high wind, flooding, rain, distance to storm track, etc.). The known and most profound impact of Hurricane Harvey was high rainfall (and subsequent flooding) [20, 21]. Therefore, we constructed small area estimates of high rainfall that are both highly spatially and temporally resolved using historic weather data compiled from regional weather stations. For Texas and Louisiana, Hurricane Harvey rainfall measurements were calculated at the 2010 ZCTA geographic centroid and assumed to be uniform for the entire ZCTA. Rainfall measurements were obtained from the National Oceanic and Atmospheric Administration's Global Historical Climatology Network [22], which collects daily measurements of rain at individual stations. Using station measurements from Texas, Louisiana, and neighboring states, we used inverse distance‐weighted interpolation to estimate daily rain at the ZCTA centroid [23]. Inverse distance‐weighted interpolation is a commonly used approach for estimating environmental exposures, where locations farther from the center receive less weight. We then applied the definition previously used by Anderson et al. for heavy rainfall exposure, where exposed ZCTAs were those that experienced greater than 75 mm (approximately 3 in.) over the 2 days before to 1 day after the closest approach of the central storm track to that ZCTA [24]. To calculate distance to central storm track measurements for each ZCTA, we used National Hurricane Center Atlantic Hurricane Databases 2 best track estimates provided within the hurricaneexposuredata package in R [25]. A measure of distance from the center of the storm track is needed to sum the cumulative precipitation over the 2 days before to 1 day after the closest approach of the central storm track to that ZCTA. The best track estimates were interpolated to 15‐min intervals over the storm's landfall period of August 25 to September 1, 2017, using the stormwindmodel package [26]. The distance from each ZCTA centroid to the nearest point of the 15‐min interpolated track was calculated, and the date and time at that point was recorded.

### 2.3 Mortality (Dependent Variable)

The primary outcome of this study was all‐cause mortality. The date of death in the MBSF was used to identify individuals who died between August 25, 2017 and August 24, 2018 [9]. Using the date that Hurricane Harvey made landfall and the date of death, we calculated the number of elapsed days since exposure up to 12 months after the hurricane.

### 2.4 Covariates

In addition to the measures used to identify relevant vulnerable sub‐groups that also served as covariates in our models, we selected several additional covariates related to exposure status and mortality risk, including sex, baseline health status, and rurality. For baseline health status, we used the Chronic Conditions Warehouse (CCW) to identify those with a subset of comorbidities in addition to those used to identify relevant sub‐groups as described above [27]. Lastly, we used ZCTA‐level Rural–Urban Commuting Area Codes to control for rurality [28].

### 2.5 Statistical Analyses

For each vulnerable population of older Americans, we calculated the following unadjusted population‐level measures: (1) mortality rates (those exposed to high rain versus those not exposed); (2) the number of deaths attributed to high rainfall exposure; (3) attributable risk (defined as the proportion of deaths attributed to exposure); and (3) relative risk. We also estimated the unadjusted cumulative incidence of mortality by exposure status for the year following Hurricane Harvey; in these analyses, groups were compared using a log‐rank test.

Cox proportional hazard models were used to adjust for covariates when estimating the individual‐level association between high rain exposure and mortality in the year following Hurricane Harvey. Models included ZCTA‐based random effects to account for clustering at the neighborhood level. Coefficients from these models were exponentiated to express the association as Hazard Ratios (HR). Models were adjusted for age (continuous), sex (male or female), and race/ethnicity (Non‐Hispanic White versus Non‐Hispanic Black, Hispanic/Latino, other), number of comorbidities (i.e., comorbidities in the CCW), and rurality (using Rural–Urban Commuting Area Codes). Separate, stratified models were run by health conditions and sociodemographics. Analyses of Medicare claims were conducted using a SAS Enterprise Guide, version 8.1 (Cary, North Carolina). Stata, version 18.0 (College Station, Texas) was used to construct cumulative incidence plots, and a combination of R 4.3.3 (Vienna, Austria) and ArcGIS Pro 3.3.1 (Redlands, California) was used to generate high rain exposure estimates and maps. Across all covariates fewer than 3% were missing and we assumed any missingness was completely at random. Because of the low amount of missingness, all analyses were based on complete case analysis. The critical α level was set to 0.05 (two‐sided).

### 2.6 Sensitivity Analyses

Our primary analyses excluded the 2.7% of individuals who relocated following Hurricane Harvey. To determine whether this impacted our results we repeated analyses including those who relocated during follow‐up. Sensitivity analyses were also conducted using the E‐value to assess the robustness of observed associations to potential unmeasured confounding [29].

## 3 Results

**FIGURE 1:**
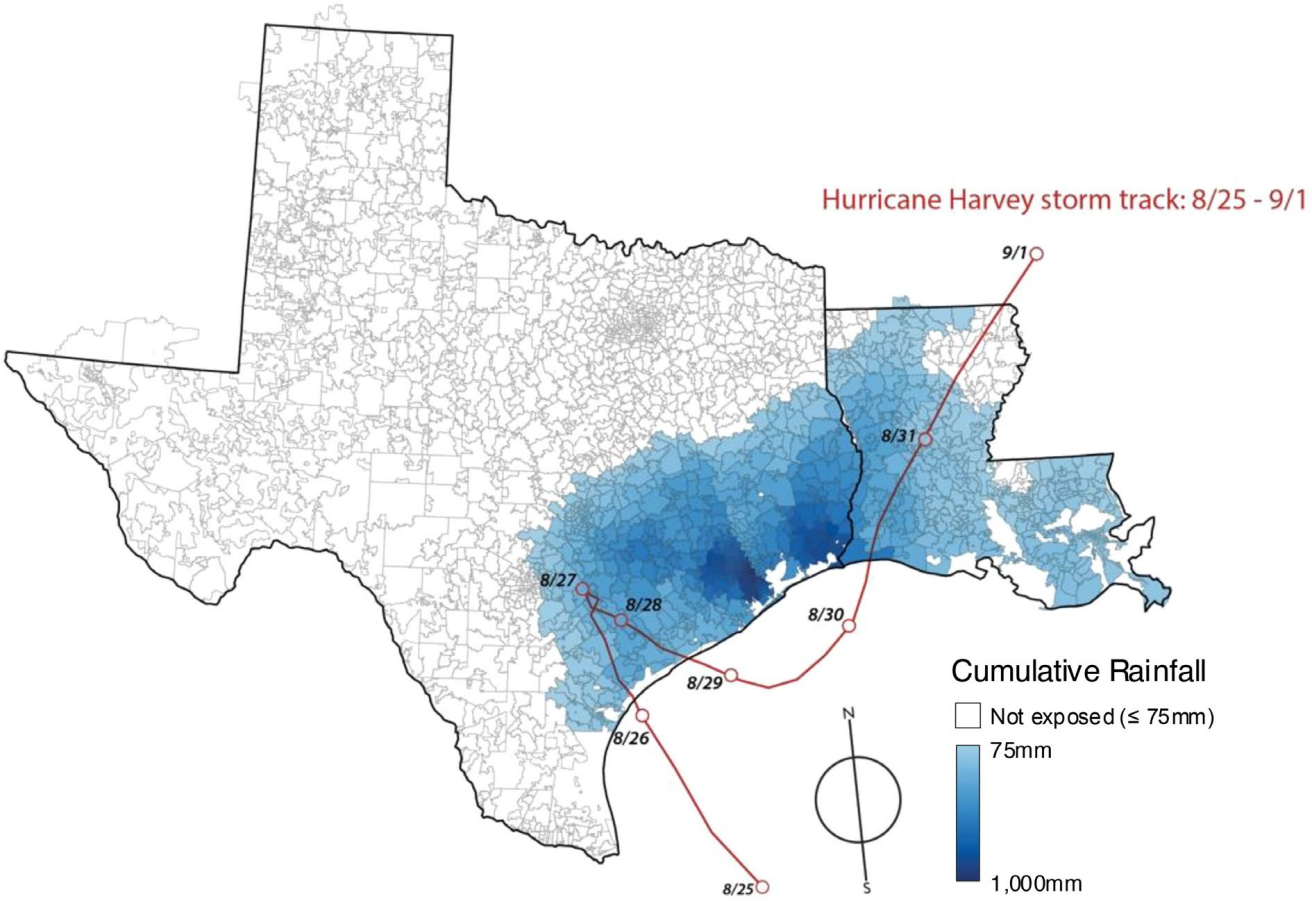
Estimated cumulative (4‐day) rainfall from hurricane harvey across ZIP code tabulation areas included in the study.

High rain‐exposed regions were concentrated within the southeastern section of Texas and more evenly distributed throughout Louisiana (Figure 1). The median estimated inches of cumulative 4‐day rainfall was 10.7 (IQR 5.8, 23.3) among areas classified as “high rain” based on the 75 mm (approximately 3 in.) threshold versus only 0.5 (IQR 0.2, 1.2) among areas that did not experience high rain, *p* < 0.0001 (Table 1).

**TABLE 1:** Characteristics of study participants according to high rain exposure status^a^.

Characteristic	Overall	By exposure status		*p* ^b^
		Exposed to high rain^a^	Not exposed to high rain	
Sample size, no.	1,781,217	828,971	952,246	
State				< 0.0001
Texas	1,468,658 (82.5)	555,467 (67.0)	913,191 (95.9)	
Louisiana	312,559 (17.5)	273,504 (33.0)	39,055 (4.1)	
Rain exposure on continuous scale				
Mean inches of rain (SD)	7.2 (10.1)	14.7 (10.7)	0.8 (0.8)	< 0.0001
Median inches of rain (IQR)	2.3 (0.4, 8.5)	10.1 (5.8, 23.3)	0.5 (0.2, 1.2)	< 0.0001
Sociodemographic characteristics				
Mean age in years (SD)	75.8 (7.3)	75.7 (7.3)	76.0 (7.4)	< 0.0001
Age category in years, no. (%)				< 0.0001
66–74	914,483 (51.3)	434,138 (52.4)	480,335 (50.4)	
75–84	613,466 (34.4)	279,915 (33.8)	333,550 (35.0)	
≥ 85	253,268 (14.2)	114,907 (13.9)	138,361 (14.5)	
Sex, no. (%)				0.145
Male	777,648 (43.7)	362,721 (43.8)	414,927 (43.6)	
Female	1,003,569 (56.3)	466,250 (56.2)	537,319 (56.4)	
Race/ethnicity, no. (%)				< 0.0001
Non‐Hispanic White	1,325,784 (74.4)	626,144 (75.5)	699,640 (73.5)	
Non‐Hispanic Black	165,227 (9.3)	102,596 (12.4)	62,631 (6.6)	
Hispanic	228,637 (12.8)	66,756 (8.1)	161,881 (17.0)	
Other	61,569 (3.5)	33,475 (4.0)	28,094 (3.0)	
Dual eligibility status, no. (%)				< 0.0001
Dual eligible	138,039 (7.7)	59,922 (7.2)	78,177 (8.2)	< 0.0001
Medicare only	1,643,178 (92.3)	769,049 (92.8)	874,129 (91.8)	
Rurality				< 0.0001
Metropolitan	1,386,561 (77.8)	671,636 (81.0)	714,925 (75.1)	
Micropolitan	211,729 (11.9)	80,978 (9.8)	130,751 (13.7)	
Small town/rural	182,846 (10.3)	76,357 (9.2)	106,489 (11.2)	
Unknown/missing	81 (0.0)	0	81 (0)	
Any hospitalization in year prior to storm				< 0.0001
No	1,504,112 (84.4)	701,903 (84.7)	802,209 (84.2)	
Yes	277,105 (15.6)	127,068 (15.3)	150,037 (15.8)	
Health condition^b^, no. with condition (%)				
ADRD	222,897 (12.5)	100,699 (12.1)	122,198 (12.8)	< 0.0001
Congestive heart failure	305,753 (17.2)	144,319 (17.4)	161,434 (17.0)	< 0.0001
Chronic pulmonary disease	247,406 (13.9)	111,028 (13.4)	136,378 (14.3)	< 0.0001
Diabetes	548,773 (30.8)	255,941 (30.9)	292,832 (30.8)	0.0766
Chronic kidney disease	488,626 (27.4)	226,091 (27.30)	262,535 (27.6)	< 0.0001
Other chronic conditions	1,557,272 (86.9)	721, 317 (87.1)	825,955 (86.7)	< 0.0001
Mean number of health conditions, SD	3.8 (2.59)	3.73 (2.57)	3.78 (2.61)	< 0.0001
Median number of health conditions, IQR	4 (2, 5)	4 (2, 5)	4 (2, 5)	< 0.0001

Abbreviations: ADRD, Alzheimer's disease and related dementias; SD, standard deviation.

^a^ High rain exposure defined as residing in area exposed to > 75 mm (approximately 3 in.).

^b^ *p* value for difference between those exposed versus not exposed to high rain. *t*‐test used for means, Mann–Whitney test for medians, and chi‐squared test for proportions.

The mean age of the sample was 75.8 years (SD 7.3); 14.2% were over the age of 85, and 7.7% were dually eligible (Table 1). Of the chronic conditions included, 12.5% had an ADRD diagnosis, and 27.4% had a CKD diagnosis. Older adults exposed to high rain, when compared to the non‐exposed population, were slightly younger (mean 75.7 versus 76.0 years, *p* < 0.001), more likely to be Non‐Hispanic White (75.5 versus 73.5%, *p* < 0.001) or Non‐Hispanic Black (NHB) adults (12.4% versus 6.6%, *p* < 0.001) relative to the non‐exposed population. Those exposed to high rain were also less likely to be dual eligible (7.2% versus 8.2%, *p* < 0.001) and more likely to reside in a metropolitan area (81% versus 75.1%, *p* < 0.001).

### 3.1 Association of High Rain Exposure and One‐Year Mortality

**FIGURE 2:**
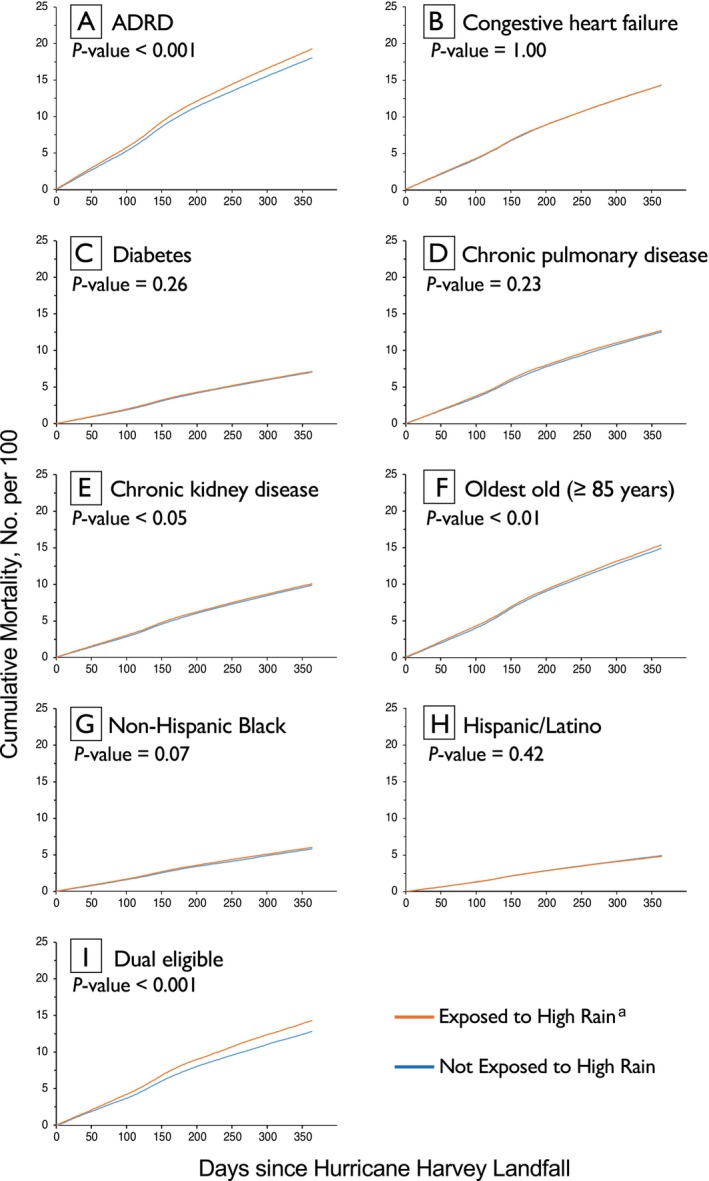
Cumulative mortality for the year following hurricane harvey by chronic health condition (A–E) and sociodemographic status (F–I). Abbreviations: ADRD, Alzheimer's disease and other related dementias; CKD, chronic kidney disease. *p* value for difference between those exposed versus not exposed to high rain based on log‐rank test. ^a^: High rain exposure defined as residing in area exposed to > 75 mm (approximately 3 in.)

**FIGURE 3:**
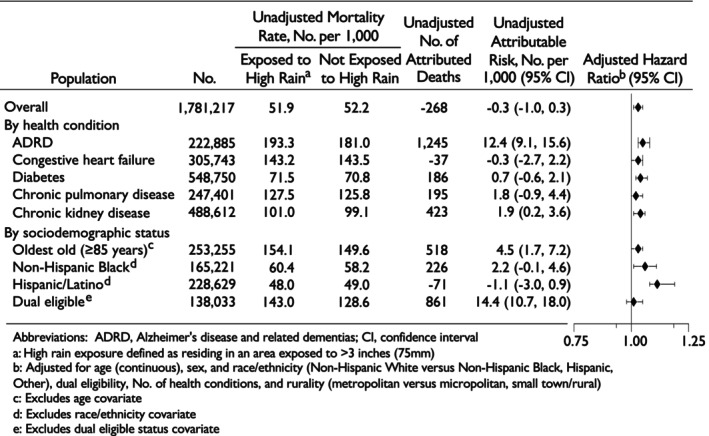
Hazard ratios for the association between high rain exposure^a^ and mortality by chronic health condition and sociodemographic status.

The unadjusted association between high rain exposure and mortality differed when stratified by those with specific health conditions and sociodemographic status (Figure 2 and Table S1). By health condition, an elevated association between high rain exposure and mortality was observed for beneficiaries with ADRD and CKD (Figure 3 and Table S2). In adjusted models, exposure to high rainfall was associated with 4% higher hazard of mortality among older adults with CKD (HR 1.04 [95% CI 1.01, 1.06]) and 5% higher hazard of mortality among older adults with ADRD (HR 1.05 [95% CI 1.03, 1.08]). Likewise, among older adults who have diabetes, high rain exposure was associated with a 4% higher hazard of mortality (HR 1.04 [95% CI: 1.02, 1.07]), and modest increased hazard for those with CHF (HR 1.03 [95% CI 1.00^+^, 1.05]), but was not significant for COPD (HR 1.02 [95% CI: 1.00^−^, 1.04]).

The association between high rain exposure and mortality also differed when stratified by sociodemographic status with the most notable elevated risk of mortality after high rain exposure among those of minoritized racial and ethnic backgrounds (Figure 3 and Table S2). In adjusted models, high rain exposure was associated with a 13% higher hazard of mortality among the Hispanic/Latino population (HR 1.13 [95% CI 1.08, 1.19]) and 6% higher among the Non‐Hispanic Black population (HR 1.06 [95% CI 1.01, 1.11]).

In unadjusted analyses, the largest number of attributed deaths was among the ADRD population—an estimated 1245 deaths in the year following Hurricane Harvey (Table S1).

Notably, attributable deaths among those with CKD (423) and those dually eligible (861) were also high. Among older adults who relocated, adjusted results did not differ in meaningful ways (Table S3). The E‐values indicated that the observed associations would require a moderately strong unmeasured confounder associated with both the exposure and the outcome to fully explain away the estimated hazard ratios (Tables S2 and S3).

## 4 Discussion

Hurricane Harvey brought extreme rainfall across Texas and Louisiana. In the year following Hurricane Harvey exposure, older adults with certain chronic conditions and racial and ethnic backgrounds faced higher risk of 1‐year mortality, along with a higher number of attributable deaths. Specifically, a high rain exposure‐mortality association (in terms of unadjusted attributable deaths) was observed among older adults with CKD and ADRD—health conditions that require regular and consistent access to healthcare and caregiving. Surprisingly, the magnitude of the association between high rainfall and mortality was similar among the oldest old and dual eligible populations to that of the overall older adult population. However, mortality was also notably elevated among the Non‐Hispanic Black and Hispanic/Latino populations. Our findings highlight that long‐term mortality hazards differ among key groups of vulnerable older adults following disasters [30, 31, 32].

Among those with ADRD, a higher mortality risk was observed, alongside 1245 attributable deaths. This corroborates past findings that living with ADRD increases the risk of mortality after hurricane exposure [33]. The number of attributable deaths in the CKD population remains high at 423 deaths as is the 4% higher HR for mortality as compared to the unexposed group. Limited past studies among hurricane‐exposed CKD populations have shown increases in short‐term mortality [15, 25]. Higher mortality was noted among Non‐Hispanic Black and Hispanic/Latino populations, calling for further investigation into the role of race and ethnicity.

Our study has notable strengths. First, while past studies of disaster‐related mortality have focused on estimating exposure at the county level and relied primarily on either FEMA‐disaster declared county designation, or distance to the central track of a storm, we constructed small area estimates of severe weather, that are both highly spatially and temporally resolved using historic weather data compiled from regional weather stations. This fine grain level of specificity allows for a more accurate understanding of mortality associated with Hurricane Harvey. Second, this study moves beyond the traditional focus on the immediate period after a disaster, by examining mortality 1 year after a catastrophic hurricane. This longer follow‐up period may reflect delayed effects that are less visible in the immediate aftermath of a disaster—such as disruptions in healthcare access or the exacerbation of chronic conditions—though further investigation is needed to better understand the mechanisms underlying long‐term mortality risk. Understanding long‐term mortality can help to guide more effective disaster management planning for clinicians and emergency managers that addresses sustained vulnerabilities in addition to acute crises. Our study contributes to this goal by reporting on one‐year mortality among different groups of older adults with pre‐existing vulnerabilities, and at a finer geographic resolution than past reports. While our findings do not directly identify modifiable factors for intervention, they underscore the need for further investigation into how systems of care for older adults with complex health and social needs can be better supported in future disasters, particularly in light of recent innovations in chronic condition care such as portable dialysis and telemedicine [26, 34]. Sustained strategies to support the health of older adults beginning in the immediate response period can better support healthy living over the longer response period. For example, among the ADRD population, use of dementia‐friendly shelters designed to support older adults and their caregivers during the immediate response period may provide the supports necessary to decrease precipitating events that lead to later adverse outcomes [35]. Further, accurately quantifying disaster mortality can also allow for prioritization of short and long‐term needs such as emergency healthcare and shelter operations as well as efficient allocation of resources and healthcare provider staffing over the extended recovery period [36].

Improved reporting measures, aggregation and integration of accurate data sources, and increased analytic efforts are needed to provide insight into mechanisms of mortality after disaster events [37, 38]. Following Hurricane Maria in 2017, researchers identified thousands of excess deaths [5, 39, 40, 41, 42], in contrast to officially reported death counts [43]. Indeed, 103 deaths in total were formally attributed to Hurricane Harvey [20], while our analysis found 3738 attributable deaths among the older adult population in the one‐year period after the hurricane, although this finding is unadjusted. Given these findings, we also emphasize the importance of longitudinal studies that assess disaster impacts over a longer time horizon.

Even with its many strengths, this study has several limitations. First, despite using highly resolved exposure measures (ZCTA rather than county‐level), some misclassification of exposure status was possible. However, this misclassification is likely non‐differential, meaning it could bias results in either direction—either toward or away from the null—by making effect estimates either too high or too low, as the true effect is unknown [24]. We acknowledge that spatial autocorrelation across neighboring ZCTAs could bias variance estimates. To address this, we included a random effect for ZCTA in all models and evaluated residual spatial autocorrelation using Moran's I. Most outcomes showed no significant residual autocorrelation; however, ADRD remained borderline significant, indicating that spatial autocorrelation may still slightly bias estimates for this outcome. Second, a limited set of sociodemographic factors were considered. For example, race categories in Medicare data are limited and may not capture the full spectrum of racial and ethnic diversity [44]. Third, our study was an observational design; therefore, despite adjusting for important covariates, we cannot rule out the possibility of residual confounding affecting our results. To address this limitation, we conducted sensitivity analyses using the E‐value to quantify the strength of an unmeasured confounder that would be required to explain away our findings. The E‐values suggested that our results were moderately sensitive. Future work is needed to solidify associations between rain exposures and increased mortality hazard. Fourth, acknowledging the potential for neighborhood selection bias, i.e., individuals who are healthier or more resourced might avoid higher‐risk flood areas, we conducted sensitivity analyses including post‐Harvey relocations, which indicated this did not substantially affect results. Finally, we selected specific types of chronic health conditions we hypothesized to be at elevated risk; however, our analyses did not account for individuals with more than one of the conditions we examined. Associations may differ among other chronic health conditions that warrant further investigation, such as cancer [45]. Future work is needed to investigate pathways from severe weather exposure to mortality among older adults with chronic health conditions, including disruptions in health care and other necessary services, to better inform interventions.

## 5 Conclusion

The development of disaster preparedness, response and recovery interventions is urgently needed to anticipate and support older Americans during times of crisis. Our findings contribute to this critical need by demonstrating populations of older Americans at greatest risk in the year following exposure to severe weather. A greater focus on healthcare resilience strategies, including improved supports for chronic disease care, as well as innovative and sustainable interventions beyond the immediate response period can support older adults in leading healthy lives after disasters in their communities.

## Author Contributions

Sue Anne Bell had full access to all the data in the study and takes responsibility for the integrity of the data and the accuracy of the analyses. Study concept and design: Matthew A. Davis and Sue Anne Bell. Acquisition of data: Sue Anne Bell, Joshua Tootoo, and Ryan Zomorrodi. Drafting of manuscript: All authors. Critical revision of the manuscript for important intellectual content: All authors. Statistical analysis: Melissa Fiffer, Jonathan Martindale, and Matthew A. Davis. Administrative, technical, or material support: Sue Anne Bell and Matthew A. Davis. Study supervision: Sue Anne Bell, Marie Lynn Miranda, and Matthew A. Davis.

## Funding

Research reported in this publication was supported by the National Institute on Aging of the National Institutes of Health under award numbers RF1AG083768 (Bell/Davis, mPI). The content is solely the responsibility of the authors and does not necessarily represent the official views of the National Institutes of Health.

## Conflicts of Interest

Dr. Matthew A. Davis reported receiving personal fees from Regional Anesthesia and Pain Medicine for statistical consultation outside the submitted work. The other authors declare no conflicts of interest.

## Supporting information


**Figure S1:** Flow diagram for the identification of study participants.
**Table S1:** Unadjusted attributable fraction and relative risk associated with exposure to high rain by chronic health condition and sociodemographic status.
**Table S2:** Unadjusted versus adjusted hazard ratios for the association between high rain exposure and mortality by chronic health condition and sociodemographic status.
**Table S3:** Hazard ratios for the association between high rain exposure and mortality by chronic health condition and sociodemographic status including study participants who relocated after hurricane harvey.
**Table S4:** Comorbidities used to define baseline health status.
