# Supplementary material for: Variation in One‐Year Mortality Following Severe Weather Exposure Among Older Americans by Chronic Health Condition and Sociodemographic Status
Source: J Am Geriatr Soc. 2025 Dec 16;74(2):377–86. doi: 10.1111/jgs.70237 (PMC12911549; doi:10.1111/jgs.70237)
Supplement: Supplementary file 1 — Figure S1: Flow diagram for the identification of study participants. Table S1: Unadjusted attributable fraction and relative risk associated with exposure to high rain by chronic health condition and sociodemographic status. Table S2: Unadjusted versus adjusted hazard ratios for the association between high rain exposure and mortality by chronic health condition and sociodemographic status. Table S3: Hazard ratios for the association between high rain exposure and mortality by chronic health condition and sociodemographic status including study participants who relocated after hurricane harvey. Table S4: Comorbidities used to define baseline health status. [file JGS-74-377-s001.pdf]

Supplemental Material

Supplementary Figure S1. Flow Diagram for the Identification of Study Participants

Supplementary Table S1. Unadjusted Attributable Fraction and Relative Risk Associated with Exposure to High Rain by Chronic Health Condition and Sociodemographic Status

Supplementary Table S2. Unadjusted versus Adjusted Hazard Ratios for the Association between High Rain Exposure and Mortality by Chronic Health Condition and Sociodemographic Status

Supplementary Table S3. Hazard Ratios for the Association between High Rain Exposure and Mortality by Chronic Health Condition and Sociodemographic Status Including Study Participants Who Relocated after Hurricane Harvey

Supplementary Table S4. Comorbidities Used to Define Baseline Health Status

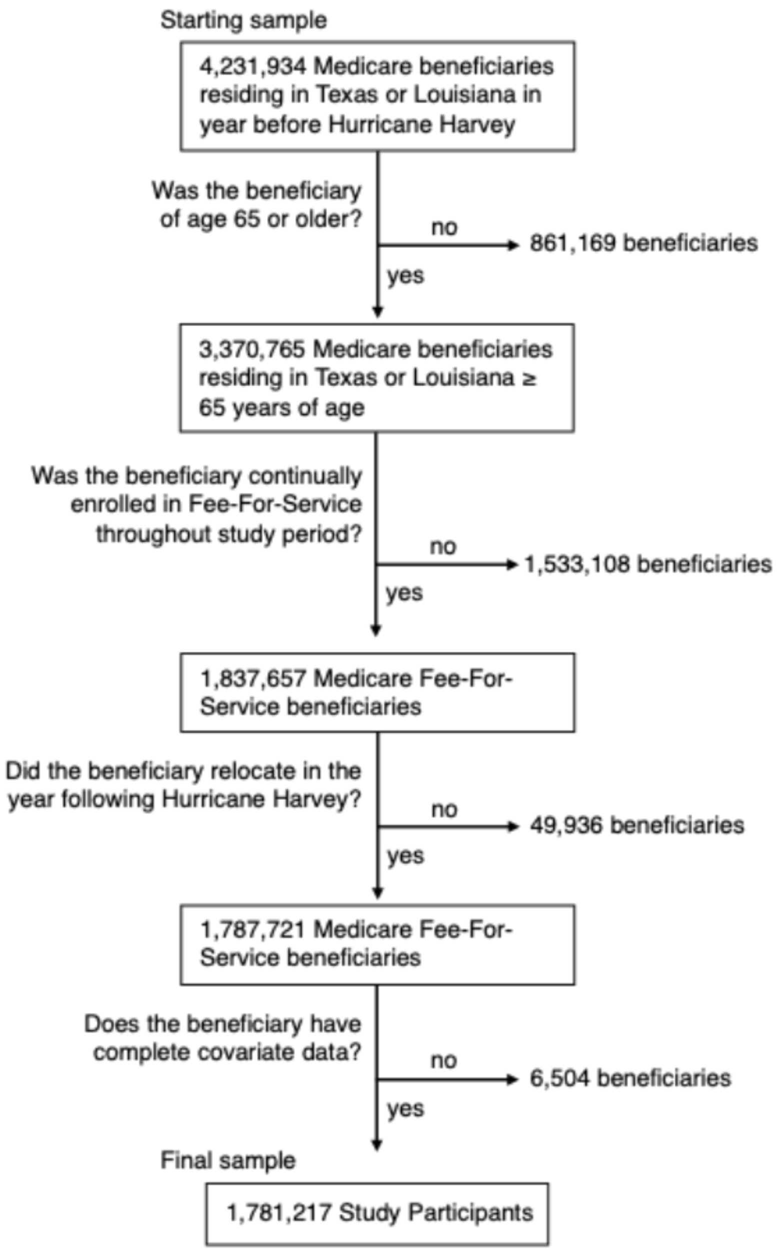
Starting sample
4,231,934 Medicare beneficiaries residing in Texas or Louisiana in year before Hurricane Harvey
Was the beneficiary of age 65 or older?
no
861,169 beneficiaries
yes
3,370,765 Medicare beneficiaries residing in Texas or Louisiana ≥ 65 years of age
Was the beneficiary continually enrolled in Fee-For-Service throughout study period?
no
1,533,108 beneficiaries
yes
1,837,657 Medicare Fee-For-Service beneficiaries
Did the beneficiary relocate in the year following Hurricane Harvey?
no
49,936 beneficiaries
yes
1,787,721 Medicare Fee-For-Service beneficiaries
Does the beneficiary have complete covariate data?
no
6,504 beneficiaries
yes
Final sample
1,781,217 Study Participants

| Population | Unadjusted Attributable fraction, % (95% CI) | Unadjusted Relative Risk (95% CI) |
|---|---|---|
| Overall | -0.6 (-1.9, 0.7) | 0.99 (0.98, 1.01) |
| By health condition | | |
| ADRD | 6.4 (4.8, 8.0) | 1.07 (1.05, 1.09) |
| Congestive heart failure | -0.2 (-1.9, 1.5) | 1.00 (0.98, 1.02) |
| Diabetes | 1.0 (-0.9, 2.9) | 1.01 (0.99, 1.03) |
| Chronic pulmonary disease | 1.4 (-0.7, 3.4) | 1.01 (0.99, 1.04) |
| Chronic Kidney Disease | 1.9 (0.2, 3.5) | 1.02 (1.00+, 1.04) |
| By sociodemographic characteristic | | |
| Oldest old (≥85 years) | 2.9 (1.1, 4.7) | 1.03 (1.01, 1.04) |
| Non-Hispanic Black | 3.6 (-0.2, 7.4) | 1.04 (1.00-, 1.08) |
| Hispanic | -2.2 (-6.4, 1.8) | 0.98 (0.94, 1.02) |
| Dual eligible older adult | 10.1 (7.6, 12.4) | 1.11 (1.08, 1.14) |

Abbreviations: ADRD, Alzheimer's disease and related dementias; CI, confidence interval

a: High rain exposure defined as residing in an area exposed to >3 inches (75mm)

| Population | Unadjusted | Adjusted[a] | E-Value[a] |
|---|---|---|---|
| Overall | 1.00 (0.98, 1.02) | 1.03 (1.01, 1.05) | 1.19 |
| By health condition | | | |
| ADRD | 1.07 (1.05, 1.1) | 1.05 (1.03, 1.08) | 1.23 |
| Congestive heart failure | 1.00 (0.98, 1.02) | 1.03 (1.00+, 1.05) | 1.19 |
| Diabetes | 1.01 (0.99, 1.04) | 1.04 (1.02, 1.07) | 1.25 |
| Chronic pulmonary disease | 1.02 (0.99, 1.04) | 1.02 (1.00-, 1.05) | 1.0 |
| Chronic kidney disease | 1.01 (0.99, 1.04) | 1.04 (1.01, 1.06) | 1.23 |
| By socidemographic status | | | |
| Oldest old (≥85 years)[b] | 1.03 (1.01, 1.06) | 1.03 (1.00+, 1.05) | 1.15 |
| Non-Hispanic Black[c] | 1.03 (0.98, 1.09) | 1.06 (1.01, 1.11) | 1.32 |
| Hispanic/Latino[c] | 1.00 (0.95, 1.05) | 1.13 (1.08, 1.19) | 1.52 |
| Dual eligible[d] | 0.99 (0.94, 1.04) | 1.01 (0.98, 1.05) | 1.0 |

Abbreviations: ADRD, Alzheimer's disease and related dementias; CI, confidence interval

a: Adjusted for age (continuous), sex, and race/ethnicity (Non-Hispanic White versus Non-Hispanic Black, Hispanic, Other), dual eligibility, No. of health conditions, and rurality (metropolitan versus micropolitan, small town/rural)
b: Excludes age covariate

c: Excludes race/ethnicity covariate

d: Excludes dual eligible status covariate

| Population | Adjusted[a] | E-Value[a] |
|---|---|---|
| Overall | 1.03 (1.01, 1.05) | 1.19 |
| By health condition | | |
| ADRD | 1.06 (1.03, 1.08) | 1.24 |
| Congestive heart failure | 1.03 (1.01, 1.05) | 1.20 |
| Diabetes | 1.05 (1.02, 1.07) | 1.26 |
| Chronic pulmonary disease | 1.03 (1.00, 1.05) | 1.18 |
| Chronic kidney disease | 1.04 (1.02, 1.06) | 1.24 |
| By sociodemographic status | | |
| Oldest old (≥85 years)[b] | 1.03 (1.00+, 1.05) | 1.16 |
| Non-Hispanic Black[c] | 1.06 (1.02, 1.11) | 1.32 |
| Hispanic/Latino[c] | 1.14 (1.09, 1.20) | 1.54 |
| Dual eligible[d] | 1.02 (0.98, 1.06) | 1.0 |

Abbreviations: ADRD, Alzheimer's disease and related dementias; CI, confidence interval

a: Adjusted for age (continuous), sex, and race/ethnicity (Non-Hispanic White versus Non-Hispanic Black, Hispanic, Other), dual eligibility, No. of health conditions, and rurality (metropolitan versus micropolitan, small town/rural)

b: Excludes age covariate

c: Excludes race/ethnicity covariate

d: Excludes dual eligible status covariate

| Comorbidity |
| --- |
| Arthritis |
| Alzheimer’s disease and related dementias (ADRD) |
| Asthma |
| Atrial fibrillation |
| Cancer (breast, colorectal, lung, prostate) |
| Chronic kidney disease |
| Chronic obstructive pulmonary disease (COPD) |
| Depression |
| Diabetes (DM) |
| Congestive heart failure (CHF) |
| Hyperlipidemia |
| Hypertension |
| Ischemic heart disease |
| Osteoporosis |
| Stroke |

**Note:**
Conditions were derived from the CMS Chronic Condition Warehouse indicators, following the approach in *Lochner, K.A., Goodman, R.A., Posner, S., & Parekh, A. (2013). Multiple chronic conditions among Medicare beneficiaries: state-level variations in prevalence, utilization, and cost, 2011. Medicare & Medicaid Research Review, 3(3).*
Conditions were summed for a total comorbidity count.
Subgroups defined by conditions of interest (e.g., ADRD, CKD, DM, CHF, COPD) excluded that condition from the count.
